# Characterization of a Novel *Col1a1*^G643S/+^ Osteogenesis Imperfecta Mouse Model with Insights into Skeletal Phenotype, Fragility, and Therapeutic Evaluations

**DOI:** 10.1007/s00223-024-01320-2

**Published:** 2025-01-03

**Authors:** Hiroyuki Saitou, Yasuhisa Ohata, Shinji Takeyari, Chiaki Nishizawa, Hirofumi Nakayama, Makoto Fujiwara, Yasuji Kitabatake, Takuo Kubota, Keiichi Ozono

**Affiliations:** 1 Department of Pediatrics, Osaka University Graduate School of Medicine, Suita, Japan; 2 Department of Pediatrics, ISEIKAI International General Hospital, 4-14, Minamiogi-machi, Kita-ku, Osaka, 530-0052 Japan; 3 Surgery for Oral and Maxillofacial Disease, Osaka University School of Dentistry, Suita, Japan

**Keywords:** Osteogenesis imperfecta, *Col1a1*, Glycine substitution, Mouse model, Micro-CT analysis, Three-point bending test

## Abstract

Osteogenesis imperfecta (OI) is an inheritable skeletal disorder characterized by bone fragility often caused by pathogenic variants in the *COL1A1* gene. Current OI mouse models with a glycine substitution in *Col1a1* exhibit excessive severity, thereby limiting long-term pathophysiological analysis and drug effect assessments. To address this limitation, we constructed a novel OI mouse model mimicking a patient with OI type III. This was achieved by introducing a G-to-A transversion at nucleotide position 2428 in the *Col1a1* gene via CRISPR-Cas9 technology in C57BL/6 J mice. The resulting heterozygous variant mice (*Col1a1*^G643S/+^) displayed reduced body weight and pronounced skeletal abnormalities. Micro-CT analysis at 12 weeks revealed decreased vertebral bone parameters and altered cortical bone characteristics, indicative of bone fragility. Additionally, the abnormalities of the anisotropy, complexity, connectivity, and structure of trabecular bone were revealed. A three-point bending test confirmed the fragility, with reduced displacement and fracture energy in both sexes. Furthermore, we evaluated the effect of 4-phenylbutyric acid on the bone in *Col1a1*^G643S/+^ mice at 12 weeks, observing no significant effects, likely due to the absence of collagen retention in the ER in this model. Despite being a moderate OI model, *Col1a1*^G643S/+^ mice manifest a distinct and fragile bone phenotype, making them suitable for extended studies. This model offers a valuable platform for investigating long-term pathophysiological aspects of OI and assessing the efficacy of potential therapeutic interventions.

**Supplementary Information:**

The online version contains supplementary material available at 10.1007/s00223-024-01320-2.

## Introduction

Osteogenesis imperfecta (OI) is a rare genetic skeletal disorder where pathogenic variants in genes affect the production of type I collagen, resulting in brittle bones. In 1979, Sillence et al. [1] proposed the Sillence classification of OI based on clinical features and disease severity. Type I is characterized by mild symptoms and is common, often presenting with blue sclera; Type II is identified as severe perinatal OI; Type III is marked by severe symptoms, accompanied by progressive deformities; Type IV is characterized as moderate to severe, with normal sclera [1]. Approximately 85–90% of cases are caused by pathogenic variants in the *COL1A1* or *COL1A2* genes, leading to defects in the α1 and α2 chains of type I collagen, respectively [2–4]. Glycine substitutions in type I collagen are reportedly the most frequent causative factors for severe and perinatal forms of OI, often associated with a more severe OI phenotype [4–6].

Recent studies investigated the potential of curative cell and gene therapy for OI. Several studies have highlighted using 4-phenylbutyric acid (4PBA) as a therapeutic agent for OI. Gioia et al. [7], Scheiber et al. [8], and Duran et al. [9] demonstrated that 4PBA ameliorates the skeletal OI phenotype through investigations using zebrafish (*Denio rerio*) and mouse models. In a previous study, we also revealed that 4PBA treatment ameliorates misfolding of the type I collagen helix in the extracellular matrix and improves osteoblast mineralization in patients with OI-derived fibroblasts and induced pluripotent stem cells [10].

Several mouse models of OI have been used to investigate the pathology and causes of OI, as well as its clinical therapies. One commonly used model is the oim mouse, first described in 1993. These mice present a spontaneous pathogenic variant in the type I collagen structure with a deficiency in the pro-α2(I) chain and exhibit phenotypic and biochemical features similar to those of human disease [11]. A mouse model with a glycine substitution in the collagenous domain of the type I collagen has been generated because the glycine substitution in type I collagen results in severe OI. This model, known as the Brittle (BrtlIV) mouse model, carries a heterozygous typical glycine substitution in one *Col1a1* allele (Gly349Cys) and is a knock-in murine model of OI reproducing the genetic variant found in children with type IV OI [12]. This model has been valuable for studying the effects of glycine substitutions in type I collagen and for investigating potential therapeutic approaches for OI [13]. However, evaluating late pathology and the effect of novel drugs is difficult because 40–60% of the mutant F2 progeny die within a few hours after birth from respiratory distress [13]. Furthermore, analyzing the BrtlIV mouse model can be challenging due to the variability in severity ranging from moderately severe to perinatal lethal [14, 15]. Another mouse model with a glycine substitution in *Col1a1* has been constructed, incorporating the Gly859Cys variant, and serves as a model of severe perinatal type II OI [16]. The Amish *Col1a2*^G610C/+^ (Gly610Cys) mouse model is a newer, moderate OI model currently being used in various intervention studies. A brittle phenotype characterizes this model, and despite being a moderate OI model, it maintains a distinctly brittle phenotype, as demonstrated through mechanical testing [17, 18].

We previously reported that glycine substitutions, not in *COL1A2* but in *COL1A1*, result in a more severe phenotype than those with other pathogenic variants [4]. To enable the investigation of late pathogenesis and drug effects on bone with *COL1A1* glycine substitutions, we aimed to construct a novel OI mouse model harboring a glycine substitution in one *Col1A1* allele and to evaluate the effects of 4PBA in this mouse model.

## Methods

### Animals

All experiments received approval from the Animal Care Committee at Osaka University (Approval #310,018–017). Our objective was to create a mouse model with a variant equivalent to that found in a patient with type III OI. This patient exhibited multiple fractures and blue sclera at birth. At the age of 10, her height was 108.1 cm (− 2.16 SD), and her lumbar spine bone mineral density (BMD) was 0.313 g/cm^2^ (Z-score = − 5.99). The patient carried a heterozygous variant (p.Gly821Ser) in the *COL1A1* gene, resulting in an alteration of the 643rd amino acid from glycine to serine in the helical region. Consequently, we introduced a G-to-A transversion at nucleotide 2428 in the *Col1a1* gene, mimicking the same amino acid alteration in C57BL/6 J mice using the CRISPR-Cas9 system at The Institute of Experimental Animal Sciences, Faculty of Medicine, Osaka University, Japan. In addition to c.2428G > A, we introduced c.2427 T > C and c.2433A > G alterations, which do not alter the amino acid sequence and are considered silent variant, for genotyping. For the knock-in, we designed single guide RNA (sgRNA) targeting *Col1A1* exon 36, where c.2428 is located, using CRISPOR (http://crispor.tefor.net/) and CRISPRdirect (https://crispr.dbcls.jp/). Additionally, we designed single-strand oligo DNA as a donor DNA template, incorporating the c.2428G > A variant along with the insert sequence, flanked by homology arms complementary to the target site. Microinjection of the sgRNA, Cas9 mRNA, and donor DNA into C57BL/6JJcl mouse zygotes was performed using a super electroporator (NEPA21®; NEPAGENE, Ichikawa, Japan). The electroporation reagent included 100 ng/µL of Cas9 endonuclease (Alt-R® S.p. HiFi Cas9 Nuclease 3NLS; Integrated DNA Technologies, Coralville, IA, USA), 200 ng/µL of sgRNA (Alt-R® CRISPR-Cas9 System; Integrated DNA Technologies), and 300 ng/µL of donor DNA (4 nmol Ultramer® DNA Oligos; Integrated DNA Technologies). Following overnight culture, 140 two-cell zygotes were injected into the gonaduct of five pseudo-pregnant ICR mice. We initially obtained 10 F0 mice including two male and two female knock-in mice. These heterozygous knock-in mice were then bred with wild-type C57BL/6 J mice to establish the colony. For this study, we analyzed mice from the F5 generation. The reproductive rate of this model mouse was comparable to that of wild-type C57BL/6 J mice, with litter sizes ranging from approximately six to ten pups.

For genotyping, target genes were amplified through polymerase chain reaction (PCR) using forward (5′-TCTAGGGAGACCGTGGTGAG-3′) and reverse (5′-CCAAGTCCAAGGCTATCCAA-3′) primers. Left and right femurs and whole vertebrae were harvested to analyze skeletal characteristics. After weaning at 3 weeks, all mice were housed in groups of one to three per cage, adhering to the regulations of the Animal Care Committee at Osaka University. Mice were kept under a 12 h light–dark cycle (light on from 08:00 to 20:00) with ad libitum access to food and water. The health and well-being of the animals were closely monitored by both the research team and staff of the Institute of Experimental Animal Sciences Faculty of Medicine, Osaka University. Monitoring included regular assessments of body weight (twice weekly), food and water intake, and general assessment of animal activity. We fed these mice with standard certified solid rodent diets (MF, Oriental Yeast Co., Ltd, Tokyo, Japan, Table S1). We collected blood samples via cardiac puncture at 8 weeks to measure serum levels of calcium, phosphate, alkaline phosphatase, and albumin. The laboratory analyses were conducted at Oriental Yeast Nagahama Lifescience laboratory (Shiga, Japan). After soft tissue stripping, the left femur and vertebrae were placed in 70% ethanol at 20 °C for microcomputed tomography (µCT) analysis, and the right femur was frozen in saline-soaked gauze at − 80 °C for three-point bending mechanical tests. These samples were kept from drying during the subsequent procedures without rehydration. To evaluate the growth longitudinally and analyze the young mouse bone phenotype, we analyzed 12-week-old mice, as reported by Jakobsen et al. and Kaupp et al.[19, 20]

### µCT and Architectural Analysis

µCT scans of the L5 vertebrae and left femurs from 12-week-old *Col1a1*^G643S/+^ mice and their normal littermates were conducted using a Scan Xmate-L090H system (Comscantecno Co., Ltd., Yokohama, Japan) operating at 75 kV and 100 µA X-ray source, according to the manufacture’s instructions, at Kureha Special Laboratory (Iwaki, Japan). The resolution was set to 10.944 and 15.894 µm/pixel for femurs and vertebrae, respectively. For the distal femur, trabecular bone was scanned in a region starting from 0.5 mm proximal to the distal femoral growth plate with a 2,604 µm section of bone in a proximal direction. Data quantification was performed using the TRI/3D-BON bone structure analysis software (Ratoc System Engineering Co., Ltd. Tokyo, Japan).

Trabecular bone parameters, based on the scanning charts, included bone surface/bone volume (BS/BV, /mm) and bone volume/total volume (BV/TV, %) for the analysis of the volume. Trabecular thickness (Tb. Th, µm), trabecular number (Tb. N, /mm), trabecular separation (Tb. Sp, µm), and trabecular spacing (Tb. Spac, µm) were calculated using parallel plate models. BMD (g/cm^2^) data were obtained from the frontal section image of L5 vertebrae and femora, calculated using phantoms of defined density. The fractal dimension (FD) was measured to assess the complexity of unevenness. The trabecular bone pattern factor (TBPf, /mm) was calculated as the ratio of the delta bone surface to delta bone volume (δBD/δBV). As an index of osteoporosis severity, star volume was analyzed and presented as the marrow space star volume (V*_m_. space, mm^3^) and trabecular star volume (V*_tr_, mm^3^). The Node-Sturt model was used to assess the trabecular structure and node (Nd, the joint point of more than three trabeculae or trabeculae with different widths) and terminus (Tm, the end without a joint with other trabeculae), and the joint with cortical bone (Ct) was measured. From these parameters, the number (*N*) and mean length (*E*) of the bone between Nds (NdNd), Nd and Tm (NdTm), Ct and Nd (CtNd), Ct and Tm (CtTm), Cts (CtCt), and Tms (TmTm) were evaluated. The total strut length (TSL) was also calculated, and the ratio to the total length of each parameter was evaluated as follows: total length of NdNd/TSL (%), NdTm/TSL (%), CtNd/TSL (%), CtTm/TSL (%), CtCt/TSL (%), and TmTm/TSL (%). To analyze the cortical bone at the midshaft femur, scanning started at 50% of the total femur length from the distal end and continued for 1 mm proximally.

For the cortical bone, cortical volume (Ct. V, mm^3^), bone volume (Bv, mm^3^), medullary volume (Mv, mm^3^), average cortical thickness (Ct. Th, µm), cortical bone area (Ct. Ar, mm^2^), and total pore volume (Po. V, mm^3^) were measured. The total cross-sectional area inside the periosteal envelope (Tt. Ar, mm^2^) was calculated as the total of Ct. V, Mv, and Po. V. Cortical bone density (Cort Bone Density, %) and total bone density (Total Bone Density, %) were calculated using the following formulas: (Ct. V) / (Ct. V + Po. V) and (Bv + Ct. V) / (Tt. Ar), respectively.

### Three-Point Bending Mechanical Test

Destructive three-point bending was conducted on the right femurs of 10 male and 10 female *Col1a1*^G643S/+^ mice and their littermates at 12 weeks of age using an MZ-500D system (Maruto Instrument Co., Ltd., Tokyo, Japan) at Kureha Special Laboratory. The posterior surface was subjected to tension at a vertical displacement rate of 2 mm/min, with a 6 mm distance between the lower supports. The maximum load (N) and displacement (mm) were measured from the load–displacement curve, and the stiffness (N/mm) was determined through linear regression of the initial region of the curve. The fracture energy (Nmm) was calculated by measuring the area under the load-deformation curve.

### Bone Histomorphometry

Bone histomorphometry was performed on lumber vertebrae at 8-week-old mice, including 4 WT and 3 *Col1a1*^G643S/+^ mice. Tetracycline was administered subcutaneously, followed by calcein injection 5 and 2 days before vertebrae removal. The vertebrae were fixed with 70% ethanol immediately after the sacrifice. Sagittal sections of the lumber vertebrae (L3) were prepared, and fluorescence imaging was performed at the Ito Bone Histomorphometry Institute (Niigata, Japan). All bone sections were analyzed histomorphometrically using a light microscope (BX-53, Olympus, Tokyo, Japan) with an image analyzer (CSS-840: System Supply Co., Nagano, Japan). The analysis was conducted at 400 × magnification, with histomorphometric parameters measured in an area of 0.0625 mm^2^ (0.25 × 0.25 mm). Sixteen to twenty areas were analyzed per sample.

### 4PBA Treatment

4PBA was administered following the protocol described by Duran et al. [9]. Briefly, 50 mg/day of 4PBA (OrphanPacific, Inc., Tokyo, Japan) was added to the drinking water and changed once per week over a 9-week treatment period (days 28 to 84). Before the treatment, water consumption was as follows: male and female *Col1a1*^G643S/+^ mice drank 4 and 3 mL of water per day, respectively. Therefore, 1.25 g and 1.9 g of 4PBA were dissolved in 100 mL of drinking water for male and female mice, respectively, ensuring an approximate intake of 50 mg per day per mouse. The body length of mice was measured from nose to hindquarters at each time point. No adverse events associated with the treatment were observed.

### SDS-PAGE and Immunofluorescence of Collagen From Fibroblast

Dermal fibroblasts were isolated from WT and *Col1a1*^G643S/+^ mice and cultured in Dulbecco Modified Eagle’s Medium (D-MEM) high glucose with L-glutamine, 10% fetal bovine serum (FBS), 25 U/mL penicillin, and 25 μg/mL streptomycin at 37 °C in a humidified incubator with 5% CO_2_. For collagen synthesis, fibroblasts were seeded at a predetermined density and cultured in D-MEM high glucose with L-glutamine, 2% FBS, 200 μM L-ascorbic acid phosphate magnesium salt n-hydrate (Wako, Tokyo, Japan), 25 U/mL penicillin, and 25 μg/mL streptomycin.

To analyze collagen, fibroblasts were cultured in collagen synthesis medium for 3 days. Collagen was extracted from the culture medium using pepsin digestion (0.1 mg/mL in 0.1 N HCl; Sigma, St Louis, MO) followed by salt precipitation with 1 M NaCl. The purified collagen was denatured at 95 °C for 3 min, subjected to electrophoresis on 10% polyacrylamide gel, and stained using Coomassie Brilliant Blue R-250 (Bio-Rad, Hercules, CA). The gel was subsequently destained in a solution containing 40% methanol and 10% acetic acid. Images of stained collagen were captured using ChemiDoc XRS Plus imaging system (Bio-Rad, Hercules, CA).

For Immunofluorescence, fibroblasts were cultured in the collagen synthesis medium with or without 5 mM 4-PBA (Sigma, St Louis, MO), for 4 days. After fixing with 4% paraformaldehyde and incubated with primary antibodies against type I collagen (Abcam, Cambridge, UK #ab34710) and protein disulfide isomerase (PDI: Enzo, New York, NY #ADI-SPA-891). This was followed by incubation with secondary antibodies: Alexa Fluor 488-conjugated anti-rabbit IgG (Jackson ImmunoResearch, West Grove, PA) and Alexa Fluor 647-conjugated anti-mouse IgG (Jackson ImmunoResearch, West Grove, PA). Muclei were stained with Hoechst 33,342 solution (Dojindo, Kumamoto, Japan). Fluorescence images were acquired from 25 fields per well using an automated IN Cell Analyzer 6000 microscope (GE Healthcare, Bucks, UK). Quantification of type I collagen accumulation in the ER was performed by calculating the ratio of the area of type I collagen co-localized with PDI to the total PDI-positive area, using IN Cell Investigator software (GE Healthcare, Bucks, UK).

### ER Stress Markers

Fibroblasts derived from WT and *Col1a1*^G643S/+^ mice were cultured in the collagen synthesis medium for 7 days, with or without 5 mM 4-PBA. After the culture period, total RNA was extracted using RNeasy Mini Kit (Qiagen, Hilden, Germany). cDNA was synthesized from the extracted RNA using ReverTra Ace qPCR RT Master Mix (Toyobo, Osaka, Japan) according to the manufacturer’s instructions. Real-time quantitative PCR (qPCR) was performed with specific primer sets using THUNDERBIRD SYBR qPCR Mix (Toyobo, Osaka, Japan) and QuantStudio 7 Flex Real-time PCR System (Applied Biosystems, Framingham, MA). Gene expression levels were quantified based on the 2^−ΔΔCt^ method and normalized to Gapdh expression.

### Statistical Analysis

All statistical analyses were conducted using JMP® Pro software version 17.0.0 (SAS Institute Inc., Cary, NC, USA). Comparisons of mean values were performed using Student’s *t* test or 2-way ANOVA with Tukey–Kramer post hoc analysis. In the 4PBA treatment analysis, we separated males and females and performed 2-way ANOVA among four groups: wild type with placebo, wild type with 4PBA, *Col1a1*^G643S/+^ with placebo, and *Col1a1*^G643S/+^ with 4PBA. Differences with *p*-values < 0.05 were considered statistically significant.

## Results

### ***Generation and General Characteristics of Col1a1***^***G643S/***+^***Mice***

**Fig. 1:**
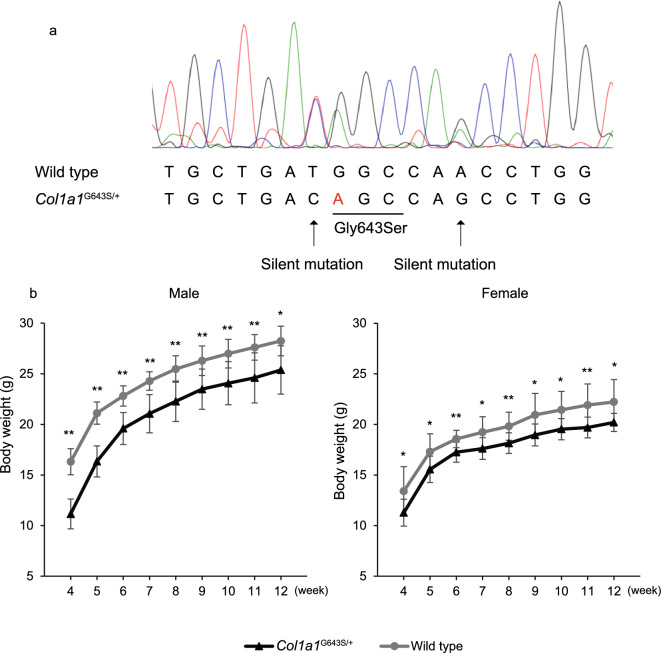
Generation and body weight measurement of *Col1a1*^G643S/+^ mice. **a** Sanger sequencing results of gDNA from wild-type and *Col1a1*^G643S/+^ mice. **b** Weight growth curve of male and female *Col1a1*^G643S/+^ mice compared with that of wild-type mice. *p < 0.05, **p < 0.01

Ten newborns were obtained, and the tail genome was isolated 3 weeks after birth. Genotyping, through PCR and direct sequencing, was performed to confirm that knock-in was successful (Fig. 1). We identified two males and two females harboring the heterogenous c.2428G > A, p.Gly810Ser variant in the *Col1a1* gene. We designated these mice *Col1a1*^G643S/+^ because the 643rd amino acid of glycine from the start of the helical region was altered to serine. We were unable to obtain any homozygous mice when heterozygotes mated, likely due to the perinatal severity of the homozygous pathogenic variant. Growth status, including body weight, was assessed in *Col1a1*^G643S/+^ mice (10 males and 10 females) and their normal littermates (wild-type [WT]: 10 males and 10 females). A growth deficiency reflected in weight gain was apparent from weaning in both male and female *Col1a1*^G643S/+^ mice, and this lower weight persisted until 12 weeks compared with that of their WT littermates. The weights of male and female *Col1a1*^G643S/+^ mice were significantly lower at 12 weeks (*p* < 0.05, Fig. 1). To assess posttranslational modifications of abnormal collagen, the mobility of collagen bands was evaluated using SDS-PAGE. Collagen derived from *Col1a1*^G643S/+^ mice exhibited slightly retarded α1 (I) and α2 (I) bands, suggesting mild overglycosylation in some α1 and α2 chains (Fig. S1). Even with the solid standard diet, the survival rate of *Col1a1*^G643S/+^ mice during and postweaning was comparable to that of WT littermates. At 8 weeks, no significant differences were detected in serum calcium, phosphate, and alkaline phosphatase levels (Table S2). Notably, no *Col1a1*^G643S/+^ mice died spontaneously until 12 weeks of age in both sexes. An apparent fracture was observed in only one *Col1a1*^G643S/+^ mouse in the left distal femur at 12 weeks; therefore, we excluded this individual from the analysis. Additionally, five male and six female *Col1a1*^G643S/+^ mice survived for over 300 days without apparent bone deformities due to fractures.

### Skeletal Characteristics

**Fig. 2:**
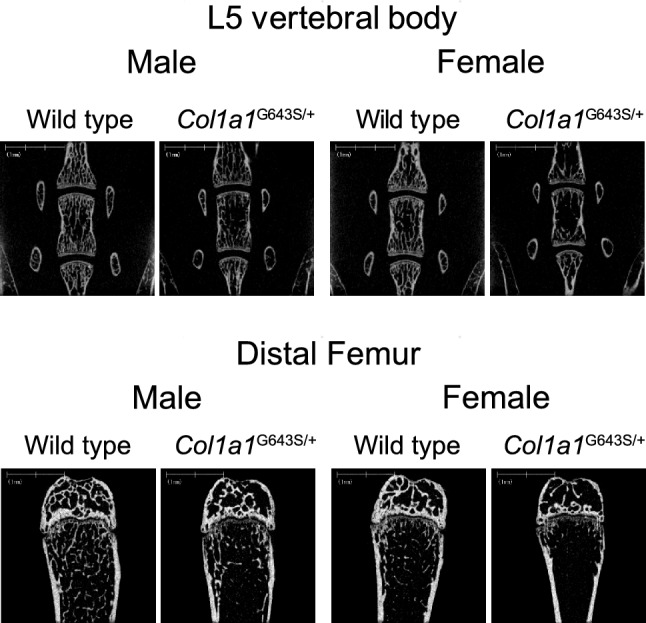
L5 vertebrae and distal femur μCT analysis of 12 weeks old mice. Shown are the cross-sectional views of 12 weeks L5 vertebrae (upper) and distal femurs (lower) from representative mice

The µCT analysis at 12 weeks revealed that the L5 vertebral BV/TV, Tb. Th, Tb. N, and BMD were all decreased and BS/BV, Tb. Sp, and Tb. Spac increased in both male and female *Col1a1*^G643S/+^ mice. At the distal femur, although BV/TV and Tb. N decreased, and Tb. Sp and Tb. Spac increased in both male and female *Col1a1*^G643S/+^ mice, no significant difference was observed in BS/BV, Tb. Th, and BMD in both sexes (Table 1). Male and female *Col1a1*^G643S/+^ mice exhibited sparse trabeculae in both the L5 vertebrae and distal femurs (Fig. 2).

**Table 1:** Trabecular bone properties in L5 vertebrae and distal femur at 12 weeks

L5 vertebral trabecular bone	Male			Female		
	Wild type (*n* = 10)	*Col1a1*^G643S/+^ (*n* = 10)	p value	Wild type (*n* = 10)	*Col1a1*^G643S/+^ (*n* = 10)	*p* value
BS/BV (/mm)	38 ± 1.2	41 ± 1.0	< 0.0001	39 ± 1.9	41 ± 2.4	0.0058
BV/TV (%)	33 ± 2.4	23 ± 3.8	< 0.0001	26 ± 2.9	15 ± 3.0	< 0.0001
Tb.Th (µm)	53 ± 1.7	49 ± 1.3	< 0.0001	52 ± 2.6	48 ± 2.8	0.0065
Tb.N (/mm)	6.2 ± 0.34	4.6 ± 0.69	< 0.0001	5.0 ± 0.4	3.1 ± 0.49	< 0.0001
Tb.Sp (µm)	110 ± 9.5	170 ± 37	< 0.0001	150 ± 17	280 ± 50	< 0.0001
Tb.Spac (µm)	160 ± 8.7	220 ± 36	< 0.0001	200 ± 16	330 ± 48	< 0.0001
BMD (g/cm^2^)	720 ± 28	670 ± 24	0.0005	710 ± 42	670 ± 33	0.0216

Femoral trabecular bone	Male			Female		
	Wild type (*n* = 10)	*Col1a1*^G643S/+^ (*n* = 10)	*p* value	Wild type (*n* = 10)	*Col1a1*^G643S/+^ (*n* = 9)	*p* value
BS/BV (/mm)	53 ± 3.0	51 ± 3.1	0.4376	58 ± 4.8	58 ± 5.0	0.7909
BV/TV (%)	17 ± 3.0	11 ± 3.7	0.0024	5.8 ± 1.9	2.8 ± 0.99	0.0007
Tb.Th (µm)	38 ± 2.2	39 ± 2.4	0.4306	35 ± 2.8	35 ± 2.9	0.7913
Tb.N (/mm)	4.3 ± 0.62	2.9 ± 0.94	0.0008	1.6 ± 0.49	0.81 ± 0.27	0.0003
Tb.Sp (µm)	200 ± 32	340 ± 140	0.0038	630 ± 220	1300 ± 530	0.0011
Tb.Spac (µm)	230 ± 31	380 ± 140	0.0036	670 ± 220	1400 ± 530	0.0011
BMD (g/cm^2^)	650 ± 36	670 ± 45	0.1889	620 ± 48	650 ± 68	0.183

Data presented as mean ± SD. *BS/BV* bone surface/bone volume, *BV/TV* bone volume/total volume, *Tb. Th* trabecular thickness, *Tb. N* trabecular number, *Tb. Sp* trabecular separation, *Tb. Spac* trabecular spacing, *BMD* bone mineral density

At the L5 vertebrae, FD decreased in *Col1a1*^G643S/+^ mice in both sexes, while it decreased only in male *Col1a1*^G643S/+^ mice at the distal femur. Although TBPf increased in *Col1a1*^G643S/+^ mice at the L5 vertebrae in both sexes, it was comparable at the distal femur in both sexes between WT and *Col1a1*^G643S/+^ mice. Star volume analysis revealed that V*m. space increased in both sexes at the L5 vertebrae and distal femur, while V*tr increased only in the L5 vertebrae in both sexes (Table 2).

**Table 2:** Static trabecular bone properties in L5 vertebrae and distal femur at 12 weeks

L5 vertebral trabecular bone	Male			Female		
	Wild type (*n* = 10)	*Col1a1*^G643S/+^ (*n* = 10)	p value	Wild type (*n* = 10)	*Col1a1*^G643S/+^ (*n* = 10)	*p* value
FD	1.4 ± 0.029	1.3 ± 0.041	< 0.0001	1.3 ± 0.03	1.2 ± 0.042	< 0.0001
TBPf (/mm)	2.9 ± 1.8	9.0 ± 2.8	< 0.0001	4.2 ± 2.3	11 ± 2.8	< 0.0001
V* m. space (mm^3^)	0.031 ± 0.011	0.12 ± 0.052	< 0.0001	0.093 ± 0.036	0.35 ± 0.095	< 0.0001
V* tr (mm^3^)	0.012 ± 0.0037	0.0071 ± 0.0024	0.0015	0.0089 ± 0.0028	0.0058 ± 0.0026	0.0196

Femoral trabecular bone	Male			Female		
	Wild type (*n* = 10)	*Col1a1*^G643S/+^ (*n* = 10)	*p* value	Wild type (*n* = 10)	*Col1a1*^G643S/+^ (*n* = 9)	*p* value
FD	1.2 ± 0.033	1.2 ± 0.04	0.0345	1.1 ± 0.042	1.1 ± 0.043	0.7202
TBPf (/mm)	14 ± 3.6	16 ± 4.0	0.1392	28 ± 5.5	27 ± 5.4	0.5205
V* m. space (mm^3^)	0.12 ± 0.055	0.57 ± 0.4	0.0023	0.83 ± 0.35	1.6 ± 0.32	< 0.0001
V* tr (mm^3^)	0.0033 ± 0.0019	0.0047 ± 0.0028	0.1817	0.002 ± 0.0012	0.0017 ± 0.00075	0.4454

Data presented as mean ± SD. *FD* fractal dimension, *TBPf* trabecular bone pattern factor, V*m. space marrow space star volume, V*_tr_: trabecular star volume

The Node-Sturt model revealed that NdNd/TSL decreased and TmTm/TSL increased in male and female *Col1a1*^G643S/+^ mice L5 vertebrae, while no difference between WT and *Col1a1*^G643S/+^ mice in the distal femur was observed. NdTm/TSL decreased only in male *Col1a1*^G643S/+^ mice L5 vertebrae. Other parameters, including CtNd/TSL, CtTm/TSL, and CtCt/TSL, did not significantly differ (Table 3 and Table S3).

**Table 3:** Node-Strut model for trabecular bone in L5 vertebrae and distal femur at 12 weeks

L5 vertebral trabecular bone	Male			Female		
	Wild type (*n* = 10)	*Col1a1*^G643S/+^ (*n* = 10)	*p* value	Wild type (*n* = 10)	*Col1a1*^G643S/+^ (*n* = 10)	*p* value
NdNd/TSL (%)	29 ± 11	8.3 ± 7.0	0.0001	16 ± 12	4.1 ± 3.1	0.0074
NdTm/TSL (%)	19 ± 9.7	11 ± 7.2	0.0664	17 ± 14	15 ± 10	0.735
CtNd/TSL (%)	18 ± 11	14 ± 13	0.5115	23 ± 11	15 ± 16	0.1769
CtTm/TSL (%)	8.9 ± 4.2	8.9 ± 7.7	0.986	8.9 ± 6.7	10 ± 8.0	0.6864
CtCt/TSL (%)	5.6 ± 6.2	7.0 ± 4.4	0.5626	8.8 ± 4.5	6.9 ± 11	0.6221
TmTm/TSL (%)	20 ± 9.9	50 ± 23	0.0014	26 ± 16	49 ± 26	0.0291
Femoral trabecular bone	Male		Female			
	Wild type (*n* = 10)	*Col1a1*^G643S/+^ (*n* = 10)	p value	Wild type (*n* = 10)	*Col1a1*^G643S/+^ (*n* = 9)	*p* value
NdNd/TSL (%)	9.8 ± 7.9	12 ± 10	0.5214	1.6 ± 2.6	1.3 ± 3.8	0.8544
NdTm/TSL (%)	22 ± 13	15 ± 9.6	0.1968	3.3 ± 5.3	5.1 ± 10	0.6167
CtNd/TSL (%)	4.0 ± 3.7	5.5 ± 5.6	0.5102	4.6 ± 6.2	0.0 ± 0.0	0.0404
CtTm/TSL (%)	1.8 ± 1.6	4.2 ± 4.4	0.1217	6.3 ± 8.7	11 ± 16	0.4171
CtCt/TSL (%)	0.67 ± 1.4	1.4 ± 2.4	0.4217	0.57 ± 1.8	5.9 ± 9.8	0.1065
TmTm/TSL (%)	62 ± 19	61 ± 16	0.9559	84 ± 16	77 ± 19	0.3883

Data presented as mean ± SD. *Nd* node, *Tm* terminus, *Ct* joint with cortical bone, *TSL* total strut length, *NdNd/TSL* ratio of the total length between nodes to total strut length, *NdTm/TSL* ratio of the total length between node and terminus to total strut length, *CtNd/TSL* ratio of the total length between joint with cortical bone and node to total strut length, *CtTm/TSL* ratio of the total length between joint with cortical bone and terminus to total strut length, *CtCt/TSL* ratio of the total length between joints with cortical bone to total strut length, *TmTm/TSL* ratio of the total length between terminuses to total strut length

**Fig. 3:**
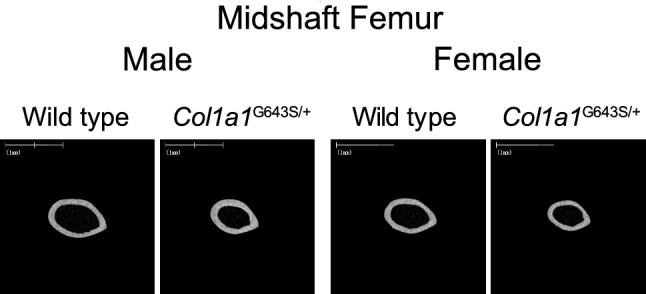
Femur midshaft μCT analysis of 12 weeks old mice. Shown are cross-sectional views of 12 weeks femurs at midshaft from representative mice

Cortical bone was thin in both sexes (Fig. 3), and Ct. V, Mv, Tt. Ar, medullary ratio, Ct. Ar, and total bone density at the midshaft femurs of *Col1a1*^G643S/+^ mice significantly decreased. The Ct. Ar/Tt. Ar was higher in male and female *Col1a1*^G643S/+^ mice compared with that in WT mice. Ct. Th, Po. V, Ct. Po, and cortical bone density in both sexes did not significantly differ (Table 4).

**Table 4:** Cortical properties in middle shaft of femur at 12 weeks

Femoral cortical bone	Male			Female		
	Wild type (*n* = 10)	*Col1a1*^G643S/+^ (*n* = 10)	*p* value	Wild type (*n* = 10)	*Col1a1*^G643S/+^ (*n* = 10)	*p* value
Ct. V (mm^3^)	0.92 ± 0.074	0.83 ± 0.068	0.0091	0.74 ± 0.052	0.68 ± 0.051	0.0164
Mv (mm^3^)	1.1 ± 0.15	0.75 ± 0.069	< 0.0001	0.86 ± 0.076	0.64 ± 0.049	< 0.0001
Tt. Ar (mm^3^)	2.0 ± 0.21	1.6 ± 0.1	< 0.0001	1.6 ± 0.11	1.3 ± 0.073	< 0.0001
medullary ratio (%)	54 ± 2.4	47 ± 3.1	< 0.0001	54 ± 1.9	48 ± 2.7	< 0.0001
Ct. Ar (mm^2^)	0.92 ± 0.074	0.83 ± 0.068	0.0091	0.74 ± 0.052	0.68 ± 0.051	0.0164
Ct. Ar/Tt. Ar (%)	46 ± 2.4	53 ± 3.0	< 0.0001	46 ± 1.9	51 ± 2.7	< 0.0001
Ct. Th (µm)	200 ± 9.8	210 ± 16	0.094	180 ± 8.5	190 ± 13	0.1965
Po. V (mm^3^)	0.000024 ± 0.000076	0.00042 ± 0.0012	0.3131	0.00013 ± 0.00024	0.00087 ± 0.0012	0.0715
Ct. Po (%)	0.0028 ± 0.0088	0.047 ± 0.13	0.313	0.017 ± 0.03	0.12 ± 0.17	0.0677
Cort Bone Density (%)	100 ± 0.0088	100 ± 0.13	0.313	100 ± 0.03	100 ± 0.17	0.0677
Total Bone density (%)	46 ± 2.4	53 ± 3.0	< 0.0001	46 ± 1.9	51 ± 2.7	< 0.0001

Data presented as mean ± SD. *Ct. V* cortical volume, *Mv* medullary volume, *Tt. Ar* total cross-section area inside the periosteal envelope, *Ct. Ar* cortical bone area, *Ct. Ar/Tt. Ar* cortical area fraction, *Ct. Th* average cortical thickness, *Po. V* total pore volume, *Ct. Po* cortical porosity

### Mechanical Properties

A three-point bending test at 12 weeks demonstrated that the maximum displacement, stiffness and fracture energy significantly decreased in male *Col1a1*^G643S/+^ mice. In females, although the maximum displacement and fracture energy were significantly reduced, as in males, no significant difference in stiffness was observed. The maximum load was comparable in both male and female *Col1a1*^G643S/+^ mice compared with that in WT mice (Table 5).

**Table 5:** Three-point bending mechanical test of femur at 12 weeks

	Male			Female		
	Wild type (n = 10)	*Col1a1*^G643S/+^ (n = 10)	*p* value	Wild type (n = 10)	*Col1a1*^G643S/+^ (n = 10)	*p* value
Maximum load (N)	18 ± 2.3	17 ± 2.2	0.4519	13 ± 1.3	13 ± 2.3	0.9141
Maximum displacemen**t** (mm)	0.88 ± 0.35	0.48 ± 0.18	0.0053	0.96 ± 0.28	0.43 ± 0.095	< 0.0001
Stiffness (N/mm)	99 ± 10	88 ± 11	0.0356	88 ± 13	75 ± 17	0.073
Fracture energy (Nmm)	11 ± 3.8	5.2 ± 1.5	0.0002	9.5 ± 2.2	3.6 ± 0.78	< 0.0001

Data presented as mean ± SD

### Bone Histomorphometry

**Fig. 4:**
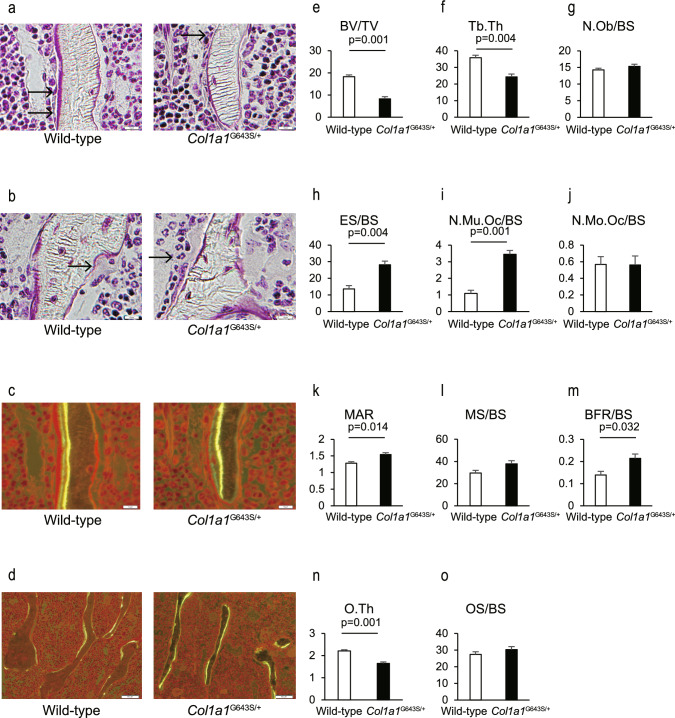
Histomorphometric analysis of lumbar vertebrae in *Col1a1*^G643S/+^ mice. **a**, **b** Representative histological images of the lumbar vertebrae (L3). Arrows indicate osteoblasts a and osteoclast (b). **c**, **d** Representative fluorescent images depicting mineral apposition rate (MAR) **c** and bone formation rate (BFR) **d**. Scale bars: 10 µm (a-c) and 100 µm (**d**). (e-o) Quantification of histomorphometric parameters of bone structure and formation: bone volume (BV)/tissue volume (TV) **e**, trabecular thickness (Tb.Th) **f**, osteoblast number (N.Ob)/ bone surface (BS) **g**, eroded surface (ES)/ BS **h**, multinucleated osteoclast number (N.Mu.Oc)/BS **i**, mononucleated osteoclast number (N.Mo.Oc)/BS **j**, MAR **k**, mineralizing surface (MS)/BS **l**, BFR/BS **m**, osteoid thickness (O.Th) **n**, and osteoid surface (OS)/BS **o**. Data are presented as mean ± SD

Consistent with the µCT analysis, *Col1a1*^G643S/+^ mice exhibited reduced BV/TV and Tb. Th. While the number of osteoblasts was comparable to WT mice, *Col1a1*^G643S/+^ mice showed an increased eroded surface and higher number of multinucleated osteoclasts. Additionally, the mineral apposition rate and bone formation rate were elevated, whereas the osteoid thickness was reduced in *Col1a1*^G643S/+^ mice (Fig. 4).

### 4PBA Effects

To assess the impact of 4PBA administration, we measured body length and weight every week during the treatment period (days 28 to 84). No significant improvement in body length and weight was observed in the 4PBA-treated group in male and female *Col1a1*^G643S/+^ mice compared with untreated *Col1a1*^G643S/+^ mice during the treatment period (Fig. S2). The µCT analysis and three-point bending test at 12 weeks showed no therapeutic effect of 4PBA administration on the trabecular properties of the L5 vertebrae and distal femurs, cortical properties in the midshaft femurs, nor the mechanical properties in the femurs of both male and female *Col1a1*^G643S/+^ mice. (Tables S4-8). No femur fractures were observed in both treated and untreated groups at 12 weeks. We measured water intake during 4PBA treatment and found a significant reduction in consumption in the 4PBA treatment group across the entire period in male and female mice (Fig. S3). While there was a trend toward reduced ER retention of type I collagen and decreased *Grp78* expression, an ER stress marker, with 4PBA treatment, these changes were not statistically significant (Fig. S4).

## Discussion

*Col1a1*^G643S/+^ mice were generated as a model for OI by introducing a glycine substitution in one *Col1a1* allele. Most trabecular and cortical bone parameters in the vertebrae and femurs of *Col1a1*^G643S/+^ mice were consistent with the bone fragility phenotype. Unexpectedly, we found no evidence supporting the effect of 4PBA. Two other OI mouse models harbor the glycine substitution in one *Col1a1* allele. Stacey et al. [16] introduced the Gly859Cys variant, establishing it as a model for type II OI owing to its severe perinatal phenotype. Another model, developed by Forlino et al. [13], features the Gly349Cys variant and is designated BrtlIV, it is considered a model for type IV OI owing to its moderate severity; nevertheless, 40–60% of newborns in this model die within a few hours after birth [13]. Forlino et al. and Bianchi et al. suggested that the heterogeneity observed in the BrtlIV mouse phenotype may be attributed to differences in their ability to adapt to cellular stress caused by mutant collagen retention [14, 15]. In contrast, *Col1a1*^G643S/+^ mice do not exhibit collagen retention or associated ER stress, resulting in a homogenous severity that facilitates more consistent studies of OI with *Col1a1* glycine substitution. The bone fragility phenotype of *Col1a1*^G643S/+^ mice in the present study, confirmed by µCT and the three-point bending test, was unexpectedly milder than those of other mouse models with a glycine substitution in one *Col1a1* allele. While data concerning genotype–phenotype relationships are limited, Rauch et al. [5] suggested that the position of the substitution may influence clinical severity. The major ligand-binding domain of COL1A1 is crucial for the interaction of collagen with other molecules, such as integrins and other extracellular matrix proteins. Variants in this domain can impact the structure and function of type I collagen, leading to the development of OI and influencing the severity of the condition. Maioli et al. [21] argued that the severity pattern due to glycine substitutions in the *COL1A1* gene showed an increase from the N- to the C-terminal end, reflecting the influence of mutation location on disease severity. Based on this evidence, the 643rd amino acid substitution from glycine to serine in the helical region was incorporated because our type III patient with OI carried a heterozygous p.Gly821Ser pathogenic variant in the *COL1A1* gene, resulting in a 643rd amino acid alteration [4, 10]. Because the proline at the 1014th position is at the C-terminal end of the COL1A1 triple helix, the 643rd glycine is situated halfway toward the C-terminal end rather than at the midpoint. The difference in genetic backgrounds between humans and mice, such as the location of the major ligand-binding domain, can influence the phenotype of the pathogenic variant. Genetic modifiers may differ between humans and mice, contributing to the variability in disease severity observed in the present study. The discrepancy in severity between our human patient with OI and *Col1a1*^G643S/+^ mice emphasizes the complexity of OI and the need to consider the interplay of genetic and environmental factors in disease phenotype [1, 3, 22].

The three-point bending test has been utilized to analyze bone fragility in OI mouse models. Carriero et al. [23] investigated the fracture toughness values using three-point bending tests on notched femurs in oim mice, revealing reduced mechanical properties in OI bone. Another study by Boraschi-Diaz et al. [24] examined bone structural properties in the *Col1a1*^Jrt/+^ mouse model of dominant OI caused by a *Col1a1* variant, and demonstrated a significant reduction in maximal load and energy until failure in young *Col1a1*^Jrt/+^ mice. Daley et al. [17] and Scheiber et al. [8] have conducted 4-point bending test on femora of *Col1a2*^G610/+^ mice, which harbor a glycine substitution in the *Col1a2* gene, and demonstrated bone fragility. In the present study, *Col1a1*^G643S/+^ mice exhibited low maximum displacement and fracture energy, representing the first study, to our knowledge, demonstrating bone fragility using the three-point bending test in an OI mouse model harboring glycine substitution in the *Col1a1* gene.

Kocijan et al. [25] analyzed a human patient with OI and demonstrated that radial and tibial BV/TV, Tb. N., and trabecular BMD decreased in type I and III–IV groups compared with those in the control group, while Tb. Th and Ct. Th significantly decreased only in type III–IV radii and type I tibias, respectively. These data are discordant with those for *Col1a1*^G643S/+^ mice in the present study. In BrtlIV mice, femoral BV/TV, Tb/Th, Tb. N., and Ct. Th decreased, whereas Tb. Sp increased at 6 months [26] However, the *Col1a1*^G643S/+^ mice in the current study exhibited no reduction in Ct. Th in both sexes. These discrepancies in each parameter can be caused by the differences in analysis methods, as we used µCT, whereas Kocijan et al. [25] and Uveges et al. [26] used high-resolution peripheral quantitative CT and bone histomorphometry, respectively.

The FD analysis of bone can be used to the anisotropy and complexity of trabecular bone structure by measuring self-homology [27]. FD has been proposed as a valuable measure for evaluating bone architecture and screening for osteoporosis, suggesting its potential as a non-invasive method for assessing structural properties in individuals with osteoporosis [28]. Recently, individuals with OI have been reported to exhibit lower FD values in condylar trabecular bone [29], and bisphosphonate treatment increases the FD in patients with OI [30]. The *Col1a1*^G643S/+^ mice in the present study demonstrated a lower L5 vertebral trabecular bone FD in both sexes. These mice can be employed for analyzing the effect of any OI treatment on the anisotropy and complexity of trabecular bone structure.

The TBPf is a histomorphometric parameter that quantitatively describes the ratio of intertrabecular connectivity, providing insights into bone microarchitecture. It has been utilized to evaluate bone properties, including bone microarchitecture assessment [31]. Although this is the first study to evaluate TBPf in OI, increased TBPf is expected in OI owing to its characterization by bone fragility and altered bone microarchitecture, leading to a more porous and less connected trabecular bone pattern. The *Col1a1*^G643S/+^ mice in the current study exhibited exceedingly high TBPf, as seen in patients with osteoporosis [32], in L5 vertebral trabecular bone but not in the femur. Collectively, these data provide insights into the characteristics of OI bone.

Star volume analysis is a stereological parameter used to describe structural changes in trabecular bone [33]. Monma et al. [34] revealed that V*m. space increased, whereas V*tr decreased in aged zebrafish, similar to the L5 vertebral trabecular bone of *Col1a1*^G643S/+^ mice. Although V*tr was comparable, V*m. space was significantly high in the femoral trabecular bone of male and female *Col1a1*^G643S/+^ mice, suggesting that star volume analysis can be employed to evaluate OI.

The node-strut model is a computational approach used to evaluate bone microarchitecture, representing the trabecular bone network as a series of nodes (intersections of trabeculae) and struts (trabeculae themselves). By analyzing the distribution and connectivity of these nodes and struts, the node-strut model provides insights into the three-dimensional microstructural properties of trabecular bone [35–37]. The node-strut model has been employed in the detection and evaluation of osteoporosis using dental panoramic radiography [38], but it has not been employed in OI. Our data demonstrating that *Col1a1*^G643S/+^ mice exhibited low NdNd/TSL and high TmTm/TSL in L5 vertebrae can serve as a standard for future OI analysis.

4PBA reportedly ameliorates cellular homeostasis in the fibroblasts of patients with OI by enhancing autophagy and stimulating protein secretion [39], positively impacting OI. Previously, we elucidated the pathogenesis wherein fibroblasts derived from patients with OI excessively produced type I collagen, which accumulated in the ER. This retention was normalized by treatment with 4PBA [10]. However, in our model mice, while 4PBA treatment slightly reduced ER retention of type I collagen and *Grp78* expression in *Col1a1*^G643S/+^, these changes were not significant. Gioia et al. [7] demonstrated that 4PBA treatment ameliorated the skeletal phenotype in a zebrafish model of OI [7]. Nevertheless, in the present study, we observed no significant effect of 4PBA on *Col1a1*^G643S/+^ mouse bones. To determine which variables most significantly affect the outcomes in the 4PBA treatment analysis, we performed multiple regression analysis. Genotype was found to have the strongest impact on the outcomes and overcomes, overriding the effect of sex and 4PBA treatment (data not shown). Further studies are needed to confirm whether *Col1a1*^G643S/+^ mouse osteoblasts lack typical pathophysiology, such as collagen overproduction or retention in ER, potentially explaining the limited effect of 4PBA on the skeletal phenotype. Complementary analysis, including liquid chromatography-mass spectrometry, would clarify if overglycosylation occurs in *Col1a1*^G643S/+^ collagen, as SDS-PAGE alone does not adequately detect such modifications.

This study had a limitation concerning the evaluation of the effect of 4PBA because we administered it to *Col1a1*^G643S/+^ mice via free drinking. Scheiber et al. [8] argued that 4PBA could reduce growth deficiency in OI by enhancing the transition of hypertrophic chondrocytes to osteoblasts, thereby improving the translocation and osteoblastic transition of hypertrophic chondrocytes in OI mouse models. In their study, Scheiber et al. [8] administered 4PBA intraperitoneally. Duran et al. [9] treated Aga2 mice with 4PBA through free drinking and observed a trend toward increased BV/TV and Tb.N., whereas no significant improvement was apparent in mechanical parameters such as ultimate load, yield load or ultimate strength. Although we attempted to administer more 4PBA in a high-dose group to enhance the effect, this experiment was halted because the mice became ill and lost body weight (data not shown). Consequently, the method we employed for 4PBA administration led to dosage uncertainty. The significant decrease in water consumption observed in the 4PBA-treated group could result in variability in the effective dosage of 4PBA. Additionally, the random assignment of mice to single or group housing may have contributed to dosage uncertainty. The intraperitoneal injection will be considered for future experiments. Another limitation of the 3-point bending test is the absence of data on diaphyseal morphology, which should be included in the calculation of femoral material properties, as recommended by the Musculoskeletal Structure and Strength Core Facility of Washington University.

In conclusion, *Col1a1*^G643S/+^ mice harboring a glycine substitution in one *Col1a1* allele were generated, exhibiting a phenotype compatible with OI. This mouse model is suitable for analyzing pathophysiology or drug effects in young adult mice, as they can survive up to 12 weeks without spontaneous fractures. This strain will contribute to determining novel therapeutic strategies based on the pathogenic variants observed in patients with OI.

## Supplementary Information

Below is the link to the electronic supplementary material.

Supplementary file1 (DOCX 16 KB)

Supplementary file2 (DOCX 18 KB)

Supplementary file3 (DOCX 16 KB)

Supplementary file4 (DOCX 19 KB)

Supplementary file5 (DOCX 19 KB)

Supplementary file6 (DOCX 18 KB)

Supplementary file7 (DOCX 26 KB)

Supplementary file8 (DOCX 18 KB)

Supplementary file9 (DOCX 17 KB)

Supplementary file10 (EPS 1380 KB)

Supplementary file11 (EPS 2928 KB)

Supplementary file12 (EPS 2719 KB)

Supplementary file13 (EPS 246675 KB)

## Declarations

### Accessibility of ***Col1a1***^G643S/+^ Mice

Researchers can access *Col1a1*^G643S/+^ mice by contacting the corresponding author and applying for export approval of living modified organisms through the Institute of Experimental Animal Sciences Faculty of Medicine, Osaka University.
