# Supplementary material for: Characterization of a Novel Col1a1G643S/+ Osteogenesis Imperfecta Mouse Model with Insights into Skeletal Phenotype, Fragility, and Therapeutic Evaluations
Source: Calcif Tissue Int. 2025 Jan 3;116(1):13. doi: 10.1007/s00223-024-01320-2 (PMC11698804; doi:10.1007/s00223-024-01320-2)
Supplement: Supplementary file 1 — Supplementary file1 (DOCX 16 KB) [file 223_2024_1320_MOESM1_ESM.docx]

**Fig. S1** SDS-PAGE analysis of type Ⅰ collagen in conditioned medium.

**Fig. S2** Effect of 4PBA on body length and weight. Body length and weight were measured weekly from 4 to 12 weeks of age. 4PBA administration was initiated from 4 weeks of age. 4PBA, 4-phenylbutyric acid. **p* < 0.05, ***p* < 0.01.

**Fig. S3** Water intake during 4PBA treatment. Measurement of water intake following the initiation of 4PBA treatment. The data represent the daily water consumption in mice treated with either a high or low dose of 4PBA and placebo. High: high-dose 4PBA treatment group, Low: low-dose 4PBA treatment group.

**Fig. S4** Intracellular localization of type Ⅰ collagen in dermal fibroblast. (a, b) Immunofluorescence analysis of dermal fibroblast from wild-type (a) and *Col1a1*^G643S/+^ (b) mice. Type Ⅰ collagen is shown in green, the endoplasmic reticulum marker protein, disulfide isomerase (PDI), is in red, and nuclei are in blue. Scale bar represents 100 µm. (c) Quantification of the colocalization of type Ⅰ collagen with PDI, expressed as the ratio of the green (type Ⅰ collagen) area merged with the red (PDI) area to the total red (PDI) area. (d, e) Real-time quantitative PCR of ER stress markers, Grp78 (d) and Chop (e). Data are presented as mean ± SD. Statistical differences were analyzed using two-way ANOVA followed by Tukey’s HSD post hoc test.
