# Supplementary material for: Characterization of a Novel Col1a1G643S/+ Osteogenesis Imperfecta Mouse Model with Insights into Skeletal Phenotype, Fragility, and Therapeutic Evaluations
Source: Calcif Tissue Int. 2025 Jan 3;116(1):13. doi: 10.1007/s00223-024-01320-2 (PMC11698804; doi:10.1007/s00223-024-01320-2)
Supplement: Supplementary file 2 — Supplementary file2 (DOCX 18 KB) [file 223_2024_1320_MOESM2_ESM.docx]

Supplemental Table 1 Mouse diet ingredients

| MF, Oriental Yeast Co., Ltd, Tokyo, Japan (per 100 g diet) | | |
| --- | --- | --- |
| Nutrient composition | Moisture (g) | 7.7 |
|  | Crude protein (g) | 23.6 |
|  | Crude fat (g) | 5.3 |
|  | Crude ash (g) | 6.1 |
|  | Crude fiber (g) | 2.9 |
|  | Nitrogen free extract (g) | 54.4 |
|  | Calories (kcal) | 360 |
| Vitamins | Vitamin A (IU) * | 2160 |
|  | Vitamin D_3_ (IU) | 158 |
|  | Vitamin E (mg) | 11.0 |
|  | Vitamin K_3_ (mg) ** | 0.04 |
|  | Vitamin B_1_ (mg) | 2.12 |
|  | Vitamin B_2_ (mg) | 1.24 |
|  | Vitamin C (mg) | 4 |
|  | Vitamin B_6_ (mg) | 0.87 |
|  | Vitamin B_12_ (µg) | 5.3 |
|  | Inositol (mg) | 578 |
|  | Biotin (µg) | 23.2 |
|  | Pantothenic acid (mg) | 2.73 |
|  | Niacin (mg) | 10.4 |
|  | Choline (g) | 0.22 |
|  | Folic acid (mg) | 0.20 |
| Minerals | Calcium (g) | 1.12 |
|  | Phosphorus (g) | 0.90 |
|  | Magnesium (g) | 0.26 |
|  | Sodium (g) | 0.21 |
|  | Potassium (g) | 0.99 |
|  | Iron (mg) | 10.9 |
|  | Aluminum (mg) | 3.1 |
|  | Copper (mg) | 0.82 |
|  | Zinc (mg) | 5.28 |
|  | Cobalt (mg) | 0.10 |
|  | Manganese (mg) | 5.89 |
|  | Ca/P | 1.24 |
|  | Ca/Mg | 4.30 |
|  | K/Na | 4.66 |
| Amino acids | Isoleucine (g) | 0.89 |
|  | Leucine (g) | 1.78 |
|  | Lysine (g) | 1.25 |
|  | Methionine (g) | 0.44 |
|  | Cystine (g) | 0.37 |
|  | Phenylalanine (g) | 1.02 |
|  | Tyrosine (g) | 0.69 |
|  | Threonine (g) | 0.92 |
|  | Tryptophan (g) | 0.29 |
|  | Valine (g) | 1.08 |
|  | Arginine (g) | 1.43 |
|  | Histidine (g) | 0.58 |
|  | Alanine (g) | 1.22 |
|  | Aspartic acid (g) | 2.14 |
|  | Glutamic acid (g) | 3.96 |
|  | Glycine (g) | 1.16 |
|  | Proline (g) | 1.27 |
|  | Serine (g) | 1.11 |
| Contaminants and others | Mercury (ppm) | 0.02 |
|  | Cadmium (ppm) | 0.07 |
|  | Lead (ppm) | 0.14 |
|  | Arsenic (as As) (ppm) | 0.3 |
|  | Selenium (ppm) | 0.41 |
|  | γ-BHC (ppm) | ND |
|  | DDT (ppm) | ND |
|  | Aldrin (ppm) | ND |
|  | Dieldrin (ppm) | ND |
|  | Heptachlor (ppm) | ND |
|  | Malathion (ppm) | 0.06 |
|  | Parathion (ppm) | ND |
|  | Aflatoxin B_1_ (ppb) | ND |
|  | Aflatoxin B_2_ (ppb) | ND |
|  | Aflatoxin G_1_ (ppb) | ND |
|  | Aflatoxin G_2_ (ppb) | ND |
|  | Polychlorobiphenyl (ppm) | ND |
|  | Estradiol (ppm) | ND |
|  | Dimethylnitrosamine (ppm) | ND |
|  | Diethylnitrosamine (ppm) | ND |
|  | Chromium (ppm) | - |

3-year average (January 1999 to December 2001)

*: Retinol, **: Amount added, ND: not detected.
