# Supplementary material for: Characterization of a Novel Col1a1G643S/+ Osteogenesis Imperfecta Mouse Model with Insights into Skeletal Phenotype, Fragility, and Therapeutic Evaluations
Source: Calcif Tissue Int. 2025 Jan 3;116(1):13. doi: 10.1007/s00223-024-01320-2 (PMC11698804; doi:10.1007/s00223-024-01320-2)
Supplement: Supplementary file 3 — Supplementary file3 (DOCX 16 KB) [file 223_2024_1320_MOESM3_ESM.docx]

Supplemental Table 2 Biochemical characteristics

|  | Wild type  (n = 4) | *Col1a1*^G643S/+^  (n = 3) | p value |
| --- | --- | --- | --- |
| serum calcium (mg/dL) | 9.88 ± 0.29 | 10.1 ± 0.033 | 0.32 |
| corrected calcium (mg/dL) | 10.6 ± 0.29 | 11.0 ± 0.46 | 0.25 |
| serum phosphate (mg/dL) | 5.68 ± 0.91 | 6.70 ± 1.25 | 0.26 |
| serum ALP (IU/L) | 119.5 ± 27.5 | 157.0 ± 66.9 | 0.35 |

Data are presented as mean ± SD. Corrected calcium levels were calculated using the following formula: serum calcium + (4 – albumin), as all albumin values were below 4.0 g/dL. ALP levels were measured using International Federation of Clinical Chemistry and Laboratory Medicine (IFCC) method. ALP: alkaline phosphatase.
