# Supplementary material for: Characterization of a Novel Col1a1G643S/+ Osteogenesis Imperfecta Mouse Model with Insights into Skeletal Phenotype, Fragility, and Therapeutic Evaluations
Source: Calcif Tissue Int. 2025 Jan 3;116(1):13. doi: 10.1007/s00223-024-01320-2 (PMC11698804; doi:10.1007/s00223-024-01320-2)
Supplement: Supplementary file 4 — Supplementary file4 (DOCX 19 KB) [file 223_2024_1320_MOESM4_ESM.docx]

Supplemental Table 3 The fundamental date of Node-Strut model for trabecular bone in L5 vertebrae and distal femur at 12 weeks

| L5 vertebral trabecular bone | Male | |  | Female | |  |
| --- | --- | --- | --- | --- | --- | --- |
|  | Wild type  (n = 10) | *Col1a1*^G643S/+^  (n = 10) | p value | Wild type  (n = 10) | *Col1a1*^G643S/+^  (n = 10) | p value |
| N. NdNd | 16 ± 8.4 | 3.3 ± 4.1 | 0.0005 | 8.6 ± 8.9 | 1.8 ± 1.8 | 0.0291 |
| N. NdTm | 9.2 ± 5.5 | 3.4 ± 2.1 | 0.0062 | 5.1 ± 4 | 3.1 ± 2.2 | 0.1809 |
| N. CtNd | 9.8 ± 6.0 | 4.3 ± 4.3 | 0.0305 | 7.4 ± 3.6 | 3.5 ± 4.5 | 0.0476 |
| N. CtTm | 2.7 ± 2.5 | 2.3 ± 2.1 | 0.7004 | 2.7 ± 0.95 | 1.8 ± 1.3 | 0.0965 |
| N. CtCt | 3.9 ± 3.7 | 2.8 ± 2.0 | 0.4151 | 4.4 ± 1.4 | 1.3 ± 1.2 | <.0001 |
| N. TmTm | 8.8 ± 3.3 | 13 ± 2.7 | 0.0052 | 7.3 ± 3 | 9.3 ± 3.4 | 0.1769 |
| E. NdNd (mm) | 0.3 ± 0.055 | 0.29 ± 0.24 | 0.8864 | 0.23 ± 0.053 | 0.11 ± 0.081 | 0.0009 |
| E. NdTm (mm) | 0.4 ± 0.19 | 0.31 ± 0.2 | 0.3179 | 0.36 ± 0.22 | 0.25 ± 0.18 | 0.2221 |
| E. CtNd (mm) | 0.29 ± 0.075 | 0.27 ± 0.17 | 0.682 | 0.33 ± 0.099 | 0.21 ± 0.17 | 0.0724 |
| E. CtTm (mm) | 0.62 ± 0.43 | 0.31 ± 0.26 | 0.0659 | 0.33 ± 0.2 | 0.28 ± 0.21 | 0.5587 |
| E. CtCt (mm) | 0.21 ± 0.13 | 0.22 ± 0.13 | 0.9314 | 0.21 ± 0.08 | 0.16 ± 0.2 | 0.474 |
| E. TmTm (mm) | 0.34 ± 0.092 | 0.31 ± 0.06 | 0.3866 | 0.34 ± 0.13 | 0.28 ± 0.085 | 0.1914 |
| TSL (mm) | 16 ± 3.4 | 8.8 ± 2.2 | <.0001 | 11 ± 3.1 | 5.8 ± 1.8 | 0.0003 |
| Femoral trabecular bone | Male | |  | Female | |  |
|  | Wild type  (n = 10) | *Col1a1*^G643S/+^  (n = 10) | p value | Wild type  (n = 10) | Wild type  (n = 9) | p value |
| N. NdNd | 7.6 ± 6.1 | 5.9 ± 6.5 | 0.5535 | 0.4 ± 0.7 | 0.33 ± 1.0 | 0.8671 |
| N. NdTm | 13 ± 9.7 | 7.1 ± 5.7 | 0.1214 | 0.8 ± 1.1 | 0.78 ± 1.6 | 0.9719 |
| N. CtNd | 2.0 ± 2.0 | 1.3 ± 1.4 | 0.3785 | 0.8 ± 1 | 0.0 ± 0.0 | 0.0332 |
| N. CtTm | 1.2 ± 1.0 | 1.1 ± 0.74 | 0.8061 | 1.0 ± 1.6 | 0.78 ± 0.83 | 0.7087 |
| N. CtCt | 0.4 ± 0.52 | 0.8 ± 1.1 | 0.3239 | 0.1 ± 0.32 | 0.44 ± 0.73 | 0.1898 |
| N. TmTm | 39 ± 7.9 | 24 ± 9.3 | 0.0009 | 19 ± 6.3 | 7.8 ± 3.4 | 0.0002 |
| E. NdNd (mm) | 0.2 ± 0.063 | 0.2 ± 0.17 | 0.969 | 0.053 ± 0.089 | 0.011 ± 0.033 | 0.1987 |
| E. NdTm (mm) | 0.27 ± 0.11 | 0.2 ± 0.076 | 0.1576 | 0.066 ± 0.1 | 0.033 ± 0.066 | 0.4337 |
| E. CtNd (mm) | 0.3 ± 0.37 | 0.3 ± 0.31 | 0.9887 | 0.13 ± 0.17 | 0.0 ± 0.0 | 0.0337 |
| E. CtTm (mm) | 0.21 ± 0.15 | 0.24 ± 0.24 | 0.7178 | 0.13 ± 0.16 | 0.12 ± 0.12 | 0.901 |
| E. CtCt (mm) | 0.085 ± 0.16 | 0.067 ± 0.12 | 0.7803 | 0.027 ± 0.085 | 0.088 ± 0.14 | 0.2648 |
| E. TmTm (mm) | 0.23 ± 0.049 | 0.22 ± 0.044 | 0.6842 | 0.15 ± 0.029 | 0.19 ± 0.091 | 0.2817 |
| TSL (mm) | 15 ± 4.6 | 9.0 ± 3.8 | 0.0031 | 3.5 ± 1.2 | 1.8 ± 0.79 | 0.0018 |

Data presented as mean ± SD. Nd: node, Tm: terminus, Ct: joint with cortical bone, N. NdNd: number of the strut between nodes, N. NdTm: number of the strut between node and terminus, N. CtNd: number of the strut between joint with cortical bone and node, N. CtCt: number of the strut between joints with cortical bone, N. TmTm: number of the strut between terminuses, E. NdNd: mean length of the strut between nodes, E. NdTm: mean length of the strut between node and terminus, E. CtNd: mean length of the strut between joint with cortical bone and node, E. CtCt: mean length of the strut between joints with cortical bone, E. TmTm: mean length of the strut between terminuses, TSL: total strut length
