# Supplementary material for: Characterization of a Novel Col1a1G643S/+ Osteogenesis Imperfecta Mouse Model with Insights into Skeletal Phenotype, Fragility, and Therapeutic Evaluations
Source: Calcif Tissue Int. 2025 Jan 3;116(1):13. doi: 10.1007/s00223-024-01320-2 (PMC11698804; doi:10.1007/s00223-024-01320-2)
Supplement: Supplementary file 5 — Supplementary file5 (DOCX 19 KB) [file 223_2024_1320_MOESM5_ESM.docx]

Supplemental Table 4 Effect of the 4PBA treatment for trabecular bone properties in L5 vertebrae and distal femur at 12 weeks

| L5 vertebral trabecular bone | Male | | | |  | Female | | | |  |
| --- | --- | --- | --- | --- | --- | --- | --- | --- | --- | --- |
|  | Wild type | | *Col1a1*^G643S/+^ | |  | Wild type | | *Col1a1*^G643S/+^ | |  |
|  | placebo  (n = 7) | 4PBA  (n = 8) | placebo  (n = 4) | 4PBA  (n = 11) | p value | placebo  (n = 5) | 4PBA  (n = 5) | placebo  (n = 6) | 4PBA  (n = 9) | p value |
| BS/BV (/mm) | 33 ± 1.1 | 36 ± 1 | 37 ± 1.4 | 39 ± 0.85 | 0.7892 | 37 ± 1.2 | 33 ± 1.2 | 39 ± 1.1 | 41 ± 0.87 | 0.3588 |
| BV/TV (%) | 34 ± 1.8 | 31 ± 1.7 | 26 ± 2.4 | 22 ± 1.5 | 0.5453 | 26 ± 1.4 | 33 ± 1.4 | 18 ± 1.3 | 16 ± 1.1 | 0.7197 |
| Tb.Th (µm) | 61 ± 1.5 | 56 ± 1.4 | 53 ± 2.0 | 52 ± 1.2 | 0.887 | 55 ± 1.6 | 61 ± 1.6 | 52 ± 1.4 | 49 ± 1.2 | 0.4685 |
| Tb.N (/mm) | 5.6 ± 0.25 | 5.5 ± 0.24 | 4.8 ± 0.34 | 4.2 ± 0.2 | 0.3626 | 4.7 ± 0.18 | 5.4 ± 0.18 | 3.4 ± 0.17 | 3.3 ± 0.13 | 0.8318 |
| Tb.Sp (µm) | 120 ± 14 | 130 ± 13 | 160 ± 18 | 200 ± 11 | 0.2373 | 160 ± 15 | 120 ± 15 | 240 ± 14 | 260 ± 11 | 0.6649 |
| Tb.Spac (µm) | 180 ± 13 | 180 ± 12 | 210 ± 17 | 250 ± 10 | 0.2228 | 210 ± 14 | 180 ± 14 | 290 ± 13 | 310 ± 11 | 0.7132 |
| BMD (g/cm^2^) | 630 ± 13 | 600 ± 12 | 580 ± 18 | 570 ± 11 | 0.9806 | 600 ± 13 | 650 ± 13 | 580 ± 12 | 580 ± 9.6 | 1.0 |
| Femoral trabecular bone | Male | | | |  | Female | | | |  |
|  | Wild type | | *Col1a1*^G643S/+^ | |  | Wild type | | *Col1a1*^G643S/+^ | |  |
|  | placebo  (n = 7) | 4PBA  (n = 8) | placebo  (n = 4) | 4PBA  (n = 11) | p value | placebo  (n = 5) | 4PBA  (n = 5) | placebo  (n = 6) | 4PBA  (n = 9) | p value |
| BS/BV (/mm) | 52 ± 2 | 56 ± 1.9 | 54 ± 2.6 | 56 ± 1.6 | 0.9634 | 58 ± 1.7 | 53 ± 1.7 | 59 ± 1.6 | 56 ± 1.3 | 0.639 |
| BV/TV (%) | 15 ± 1.6 | 14 ± 1.5 | 12 ± 2.1 | 9.4 ± 1.2 | 0.6985 | 6.3 ± 0.56 | 9.8 ± 0.56 | 3.4 ± 0.52 | 4.6 ± 0.42 | 0.2847 |
| Tb.Th (µm) | 39 ± 1.4 | 36 ± 1.3 | 37 ± 1.9 | 36 ± 1.1 | 0.9918 | 35 ± 1.1 | 38 ± 1.1 | 34 ± 0.97 | 36 ± 0.79 | 0.589 |
| Tb.N (/mm) | 3.7 ± 0.35 | 3.8 ± 0.33 | 3.3 ± 0.46 | 2.6 ± 0.28 | 0.5529 | 1.8 ± 0.15 | 2.6 ± 0.15 | 0.99 ± 0.14 | 1.3 ± 0.11 | 0.3598 |
| Tb.Sp (µm) | 260 ± 44 | 230 ± 42 | 290 ± 59 | 400 ± 35 | 0.3778 | 550 ± 93 | 350 ± 93 | 1100 ± 85 | 760 ± 70 | 0.0548 |
| Tb.Spac (µm) | 300 ± 44 | 270 ± 41 | 330 ± 58 | 440 ± 35 | 0.3731 | 590 ± 93 | 390 ± 93 | 1100 ± 85 | 800 ± 69 | 0.0561 |
| BMD (g/cm^2^) | 610 ± 20 | 560 ± 19 | 580 ± 27 | 590 ± 16 | 0.9409 | 570 ± 26 | 570 ± 26 | 580 ± 24 | 560 ± 19 | 0.937 |

Data presented as mean ± SD. BS/BV: bone surface/bone volume, BV/TV: bone volume/total volume, Tb. Th: trabecular thickness, Tb. N: trabecular number, Tb. Sp: trabecular separation, Tb. Spac: trabecular spacing, BMD: bone mineral density, p-value present the data between *Col1a1*^G643S/+^ placebo and 4PBA treatment analyzed by ANOVA followed by Tukey-Kramer post hoc test.
