# Supplementary material for: Characterization of a Novel Col1a1G643S/+ Osteogenesis Imperfecta Mouse Model with Insights into Skeletal Phenotype, Fragility, and Therapeutic Evaluations
Source: Calcif Tissue Int. 2025 Jan 3;116(1):13. doi: 10.1007/s00223-024-01320-2 (PMC11698804; doi:10.1007/s00223-024-01320-2)
Supplement: Supplementary file 6 — Supplementary file6 (DOCX 18 KB) [file 223_2024_1320_MOESM6_ESM.docx]

Supplemental Table 5 Effect of the 4PBA treatment for static trabecular bone properties in L5 vertebrae and distal femur at 12 weeks

| L5 vertebral trabecular bone | Male | | | |  | Female | | | |  |
| --- | --- | --- | --- | --- | --- | --- | --- | --- | --- | --- |
|  | Wild type | | *Col1a1*^G643S/+^ | |  | Wild type | | *Col1a1*^G643S/+^ | |  |
|  | placebo  (n = 7) | 4PBA  (n = 8) | placebo  (n = 4) | 4PBA  (n = 11) | p value | placebo  (n = 5) | 4PBA  (n = 5) | placebo  (n = 6) | 4PBA  (n = 9) | p value |
| FD | 1.4 ± 0.016 | 1.3 ± 0.015 | 1.3 ± 0.021 | 1.3 ± 0.012 | 0.2562 | 1.3 ± 0.014 | 1.3 ± 0.014 | 1.2 ± 0.013 | 1.2 ± 0.010 | 0.3847 |
| TBPf (/mm) | 1.5 ± 1.0 | 4.0 ± 0.97 | 6.1 ± 1.4 | 8.7 ± 0.83 | 0.4079 | 5.1 ± 1.2 | 1.4 ± 1.2 | 9.2 ± 1.1 | 12 ± 0.89 | 0.1702 |
| V* m. space (mm^3^) | 0.053 ± 0.028 | 0.047 ± 0.027 | 0.10 ± 0.038 | 0.18 ± 0.023 | 0.2885 | 0.14 ± 0.058 | 0.072 ± 0.058 | 0.32 ± 0.053 | 0.37 ± 0.043 | 0.8937 |
| V* tr (mm^3^) | 0.014 ± 0.0014 | 0.011 ± 0.0013 | 0.0086 ± 0.0018 | 0.0097 ± 0.0011 | 0.9549 | 0.0096 ± 0.0034 | 0.014 ± 0.0034 | 0.0079 ± 0.0031 | 0.0094 ± 0.0025 | 0.9804 |
| Femoral trabecular bone | Male | | | |  | Female | | | |  |
|  | Wild type | | *Col1a1*^G643S/+^ | |  | Wild type | | *Col1a1*^G643S/+^ | |  |
|  | placebo  (n = 7) | 4PBA  (n = 8) | placebo  (n = 4) | 4PBA  (n = 11) | p value | placebo  (n = 5) | 4PBA  (n = 5) | placebo  (n = 6) | 4PBA  (n = 9) | p value |
| FD | 1.2 ± 0.016 | 1.2 ± 0.015 | 1.2 ± 0.021 | 1.2 ± 0.013 | 0.4558 | 1.1 ± 0.020 | 1.2 ± 0.020 | 1.1 ± 0.018 | 1.2 ± 0.015 | 0.1573 |
| TBPf (/mm) | 17 ± 2.0 | 19 ± 1.9 | 18 ± 2.6 | 22 ± 1.6 | 0.7004 | 26 ± 2.2 | 19 ± 2.2 | 25 ± 2 | 22 ± 1.6 | 0.649 |
| V* m. space (mm^3^) | 0.22 ± 0.10 | 0.18 ± 0.098 | 0.29 ± 0.14 | 0.62 ± 0.084 | 0.2132 | 0.76 ± 0.14 | 0.54 ± 0.14 | 1.6 ± 0.13 | 1.4 ± 0.11 | 0.5283 |
| V* tr (mm^3^) | 0.0051 ± 0.00087 | 0.0019 ± 0.00081 | 0.0038 ± 0.0012 | 0.0027 ± 0.00069 | 0.8427 | 0.0017 ± 0.00065 | 0.0034 ± 0.00065 | 0.0017 ± 0.00059 | 0.0023 ± 0.00048 | 0.8519 |

Data presented as mean ± SD. FD: fractal dimension, TBPf: trabecular bone pattern factor, V*m. space: marrow space star volume, V*_tr_: trabecular star volume, p-value present the data between *Col1a1*^G643S/+^ placebo and 4PBA treatment analyzed by ANOVA followed by Tukey-Kramer post hoc test.
