# Supplementary material for: Characterization of a Novel Col1a1G643S/+ Osteogenesis Imperfecta Mouse Model with Insights into Skeletal Phenotype, Fragility, and Therapeutic Evaluations
Source: Calcif Tissue Int. 2025 Jan 3;116(1):13. doi: 10.1007/s00223-024-01320-2 (PMC11698804; doi:10.1007/s00223-024-01320-2)
Supplement: Supplementary file 7 — Supplementary file7 (DOCX 26 KB) [file 223_2024_1320_MOESM7_ESM.docx]

Supplemental Table 6 Effect of the 4PBA treatment for Node-Strut model for trabecular bone in L5 vertebrae and distal femur at 12 weeks

| L5 vertebral trabecular bone | Male | | | |  | Female | | | |  |
| --- | --- | --- | --- | --- | --- | --- | --- | --- | --- | --- |
|  | Wild type | | *Col1a1*^G643S/+^ | |  | Wild type | | *Col1a1*^G643S/+^ | |  |
|  | placebo  (n = 7) | 4PBA  (n = 8) | placebo  (n = 4) | 4PBA  (n = 11) | p value | placebo  (n = 5) | 4PBA  (n = 5) | placebo  (n = 6) | 4PBA  (n = 9) | p value |
| N. NdNd | 23 ± 2.6 | 16 ± 2.4 | 6.5 ± 3.5 | 5.2 ± 2.1 | 0.9878 | 10 ± 2.3 | 23 ± 2.3 | 2.7 ± 2.1 | 2.1 ± 1.7 | 0.997 |
| N. NdTm | 15 ± 1.8 | 13 ± 1.7 | 5.8 ± 2.3 | 7.0 ± 1.4 | 0.9674 | 9 ± 1.6 | 11 ± 1.6 | 4.7 ± 1.5 | 3.2 ± 1.2 | 0.877 |
| N. CtNd | 11 ± 1.5 | 8.4 ± 1.4 | 5.5 ± 2 | 3.5 ± 1.2 | 0.8286 | 7.2 ± 1.3 | 13 ± 1.3 | 3.2 ± 1.2 | 1.8 ± 1.0 | 0.8147 |
| N. CtTm | 2.3 ± 0.63 | 2.4 ± 0.59 | 2.0 ± 0.84 | 2.0 ± 0.51 | 1 | 3 ± 0.57 | 1.8 ± 0.57 | 1.2 ± 0.52 | 1.9 ± 0.43 | 0.7117 |
| N. CtCt | 2.7 ± 0.6 | 2.6 ± 0.56 | 3.3 ± 0.79 | 1.6 ± 0.48 | 0.3179 | 3.2 ± 0.82 | 3.2 ± 0.82 | 2.3 ± 0.75 | 1.6 ± 0.61 | 0.8528 |
| N. TmTm | 6.9 ± 1.4 | 11 ± 1.3 | 15 ± 1.9 | 13 ± 1.1 | 0.9204 | 9.4 ± 1.5 | 6.0 ± 1.5 | 12 ± 1.3 | 13 ± 1.1 | 0.7834 |
| E. NdNd (mm) | 0.3 ± 0.035 | 0.24 ± 0.033 | 0.25 ± 0.047 | 0.27 ± 0.028 | 0.9685 | 0.3 ± 0.061 | 0.22 ± 0.061 | 0.17 ± 0.056 | 0.16 ± 0.046 | 0.9995 |
| E. NdTm (mm) | 0.37 ± 0.03 | 0.32 ± 0.028 | 0.34 ± 0.04 | 0.28 ± 0.024 | 0.5943 | 0.32 ± 0.06 | 0.39 ± 0.06 | 0.2 ± 0.054 | 0.23 ± 0.044 | 0.9831 |
| E. CtNd (mm) | 0.24 ± 0.037 | 0.25 ± 0.034 | 0.3 ± 0.049 | 0.22 ± 0.029 | 0.5484 | 0.24 ± 0.05 | 0.26 ± 0.05 | 0.13 ± 0.046 | 0.12 ± 0.038 | 0.9936 |
| E. CtTm (mm) | 0.28 ± 0.071 | 0.36 ± 0.066 | 0.39 ± 0.094 | 0.28 ± 0.057 | 0.7524 | 0.27 ± 0.11 | 0.22 ± 0.11 | 0.26 ± 0.1 | 0.45 ± 0.085 | 0.497 |
| E. CtCt (mm) | 0.27 ± 0.058 | 0.26 ± 0.054 | 0.17 ± 0.076 | 0.24 ± 0.046 | 0.8678 | 0.13 ± 0.059 | 0.20 ± 0.059 | 0.18 ± 0.054 | 0.25 ± 0.044 | 0.7768 |
| E. TmTm (mm) | 0.31 ± 0.034 | 0.33 ± 0.031 | 0.38 ± 0.044 | 0.30 ± 0.027 | 0.4833 | 0.32 ± 0.04 | 0.31 ± 0.04 | 0.25 ± 0.036 | 0.23 ± 0.03 | 0.9753 |
| TSL (mm) | 18 ± 1.2 | 15 ± 1.1 | 12 ± 1.6 | 9.6 ± 0.95 | 0.6212 | 12 ± 1.0 | 16 ± 1.0 | 6.8 ± 0.95 | 6.0 ± 0.77 | 0.9101 |
| NdNd/TSL (%) | 35 ± 2.8 | 23 ± 2.7 | 14 ± 3.8 | 13 ± 2.3 | 0.9891 | 23 ± 4.3 | 31 ± 4.3 | 8.5 ± 3.9 | 7.4 ± 3.2 | 0.9965 |
| NdTm/TSL (%) | 30 ± 4.4 | 28 ± 4.1 | 15 ± 5.8 | 20 ± 3.5 | 0.8788 | 23 ± 5.5 | 27 ± 5.5 | 17 ± 5.0 | 14 ± 4.1 | 0.96 |
| CtNd/TSL (%) | 14 ± 2.6 | 14 ± 2.5 | 12 ± 3.5 | 8.0 ± 2.1 | 0.7448 | 15 ± 3.2 | 21 ± 3.2 | 8.1 ± 2.9 | 5.1 ± 2.4 | 0.8558 |
| CtTm/TSL (%) | 4.4 ± 1.8 | 6.1 ± 1.7 | 6.4 ± 2.4 | 7.0 ± 1.4 | 0.996 | 7.6 ± 3.6 | 2.6 ± 3.6 | 10 ± 3.3 | 12 ± 2.7 | 0.9559 |
| CtCt/TSL (%) | 4.4 ± 2 | 4.2 ± 1.8 | 5.1 ± 2.6 | 6.2 ± 1.6 | 0.9831 | 5.7 ± 2.8 | 4.0 ± 2.8 | 9.5 ± 2.6 | 6.4 ± 2.1 | 0.79 |
| TmTm/TSL (%) | 12 ± 6.4 | 24 ± 6.0 | 48 ± 8.5 | 46 ± 5.1 | 0.9993 | 26 ± 8.5 | 13 ± 8.5 | 47 ± 7.8 | 55 ± 6.3 | 0.8472 |
| Femoral trabecular bone | Male | | | |  | Female | | | |  |
|  | Wild type | | *Col1a1*^G643S/+^ | |  | Wild type | | *Col1a1*^G643S/+^ | |  |
|  | placebo  (n = 7) | 4PBA  (n = 8) | placebo  (n = 4) | 4PBA  (n = 11) | p value | placebo  (n = 5) | 4PBA  (n = 5) | placebo  (n = 6) | 4PBA  (n = 9) | p value |
| N. NdNd | 8.3 ± 3.0 | 1.8 ± 2.9 | 6.8 ± 4.0 | 3.6 ± 2.4 | 0.9107 | 1.4 ± 0.91 | 4.6 ± 0.91 | 0.83 ± 0.83 | 1.4 ± 0.68 | 0.9404 |
| N. NdTm | 6.4 ± 2.2 | 7.1 ± 2.1 | 3.8 ± 3.0 | 3.7 ± 1.8 | 1 | 2 ± 0.81 | 5.4 ± 0.81 | 0.33 ± 0.74 | 0.89 ± 0.61 | 0.9371 |
| N. CtNd | 2.6 ± 0.65 | 0.88 ± 0.61 | 2.3 ± 0.87 | 0.45 ± 0.52 | 0.3078 | 0.6 ± 0.44 | 1.4 ± 0.44 | 0.17 ± 0.40 | 0.89 ± 0.33 | 0.5132 |
| N. CtTm | 1.9 ± 0.44 | 1.0 ± 0.41 | 0.5 ± 0.58 | 0.45 ± 0.35 | 0.9999 | 0.4 ± 0.33 | 1.4 ± 0.33 | 0.33 ± 0.31 | 0.33 ± 0.25 | 1 |
| N. CtCt | 0.86 ± 0.24 | 0.63 ± 0.23 | 0.0± 0.32 | 0.27 ± 0.2 | 0.8878 | 0.4 ± 0.34 | 1.2 ± 0.34 | 0.17 ± 0.31 | 0.22 ± 0.26 | 0.999 |
| N. TmTm | 33 ± 4 | 39 ± 3.8 | 31 ± 5.3 | 26 ± 3.2 | 0.885 | 21 ± 2.8 | 20 ± 2.8 | 8.5 ± 2.5 | 11 ± 2.1 | 0.8674 |
| E. NdNd (mm) | 0.15 ± 0.043 | 0.16 ± 0.04 | 0.2 ± 0.057 | 0.072 ± 0.034 | 0.2739 | 0.14 ± 0.049 | 0.16 ± 0.049 | 0.17 ± 0.045 | 0.055 ± 0.037 | 0.2166 |
| E. NdTm (mm) | 0.22 ± 0.057 | 0.18 ± 0.053 | 0.36 ± 0.075 | 0.12 ± 0.045 | 0.0464 | 0.11 ± 0.048 | 0.28 ± 0.048 | 0.063 ± 0.044 | 0.10 ± 0.036 | 0.8877 |
| E. CtNd (mm) | 0.2 ± 0.082 | 0.18 ± 0.077 | 0.41 ± 0.11 | 0.029 ± 0.065 | 0.0284 | 0.058 ± 0.046 | 0.14 ± 0.046 | 0.013 ± 0.042 | 0.063 ± 0.034 | 0.7887 |
| E. CtTm (mm) | 0.24 ± 0.053 | 0.089 ± 0.05 | 0.057 ± 0.07 | 0.066 ± 0.042 | 0.9994 | 0.026 ± 0.048 | 0.19 ± 0.048 | 0.063 ± 0.044 | 0.052 ± 0.036 | 0.9969 |
| E. CtCt (mm) | 0.085 ± 0.037 | 0.086 ± 0.035 | 0.0 ± 0.050 | 0.061 ± 0.03 | 0.7152 | 0.03 ± 0.049 | 0.098 ± 0.049 | 0.056 ± 0.044 | 0.048 ± 0.036 | 0.9988 |
| E. TmTm (mm) | 0.22 ± 0.016 | 0.19 ± 0.015 | 0.20 ± 0.021 | 0.19 ± 0.013 | 0.9932 | 0.15 ± 0.016 | 0.16 ± 0.016 | 0.14 ± 0.015 | 0.19 ± 0.012 | 0.1425 |
| TSL (mm) | 12 ± 1.6 | 9.9 ± 1.5 | 9.1 ± 2.1 | 6.5 ± 1.2 | 0.6795 | 4.1 ± 0.48 | 6.2 ± 0.48 | 1.7 ± 0.44 | 2.6 ± 0.36 | 0.4232 |
| NdNd/TSL (%) | 8.8 ± 3.5 | 3.1 ± 3.3 | 11 ± 4.7 | 6.6 ± 2.8 | 0.8506 | 5.8 ± 4.7 | 11 ± 4.7 | 14 ± 4.3 | 6.2 ± 3.5 | 0.4837 |
| NdTm/TSL (%) | 9.7 ± 3.7 | 15 ± 3.4 | 13 ± 4.9 | 8.6 ± 2.9 | 0.8805 | 8.8 ± 5.7 | 23 ± 5.7 | 7.9 ± 5.2 | 5.5 ± 4.2 | 0.9847 |
| CtNd/TSL (%) | 4.2 ± 1.6 | 3.0 ± 1.5 | 9.3 ± 2.1 | 0.56 ± 1.3 | 0.0082 | 1.8 ± 2.1 | 5.3 ± 2.1 | 0.43 ± 1.9 | 4.2 ± 1.6 | 0.4236 |
| CtTm/TSL (%) | 5.7 ± 1.3 | 1.7 ± 1.3 | 0.5 ± 1.8 | 1.8 ± 1.1 | 0.9202 | 1.2 ± 2.2 | 6.3 ± 2.2 | 3.7 ± 2.0 | 2.2 ± 1.7 | 0.9411 |
| CtCt/TSL (%) | 1.0 ± 0.5 | 1.0 ± 0.46 | 0.0 ± 0.66 | 0.93 ± 0.4 | 0.6199 | 1.3 ± 2.9 | 3.7 ± 2.9 | 4.4 ± 2.7 | 2.2 ± 2.2 | 0.9238 |
| TmTm/TSL (%) | 71 ± 6.7 | 76 ± 6.3 | 66 ± 8.9 | 81 ± 5.4 | 0.4828 | 81 ± 9.5 | 51 ± 9.5 | 69 ± 8.7 | 80 ± 7.1 | 0.8002 |

Data presented as mean ± SD. Nd: node, Tm: terminus, Ct: joint with cortical bone, N. NdNd: number of the strut between nodes, N. NdTm: number of the strut between node and terminus, N. CtNd: number of the strut between joint with cortical bone and node, N. CtCt: number of the strut between joints with cortical bone, N. TmTm: number of the strut between terminuses, E. NdNd: mean length of the strut between nodes, E. NdTm: mean length of the strut between node and terminus, E. CtNd: mean length of the strut between joint with cortical bone and node, E. CtCt: mean length of the strut between joints with cortical bone, E. TmTm: mean length of the strut between terminuses, TSL: total strut length, NdNd/TSL: ratio of the total length between nodes to total strut length, NdTm/TSL: ratio of the total length between node and terminus to total strut length, CtNd/TSL: ratio of the total length between joint with cortical bone and node to total strut length, CtTm/TSL: ratio of the total length between joint with cortical bone and terminus to total strut length, CtCt/TSL: ratio of the total length between joints with cortical bone to total strut length, TmTm/TSL: ratio of the total length between terminuses to total strut length, p-value present the data between *Col1a1*^G643S/+^ placebo and 4PBA treatment analyzed by ANOVA followed by Tukey-Kramer post hoc test.
