# Supplementary material for: Characterization of a Novel Col1a1G643S/+ Osteogenesis Imperfecta Mouse Model with Insights into Skeletal Phenotype, Fragility, and Therapeutic Evaluations
Source: Calcif Tissue Int. 2025 Jan 3;116(1):13. doi: 10.1007/s00223-024-01320-2 (PMC11698804; doi:10.1007/s00223-024-01320-2)
Supplement: Supplementary file 8 — Supplementary file8 (DOCX 18 KB) [file 223_2024_1320_MOESM8_ESM.docx]

Supplemental Table 7 Effect of the 4PBA treatment for cortical properties in middle shaft of femur at 12

| Femoral cortical bone | Male | | | |  | Female | | | |  |
| --- | --- | --- | --- | --- | --- | --- | --- | --- | --- | --- |
|  | Wild type | | *Col1a1*^G643S/+^ | |  | Wild type | | *Col1a1*^G643S/+^ | |  |
|  | placebo  (n = 7) | 4PBA  (n = 8) | placebo  (n = 4) | 4PBA  (n = 11) | p value | placebo  (n = 5) | 4PBA  (n = 5) | placebo  (n = 6) | 4PBA  (n = 9) | p value |
| Cv (mm^2^) | 0.82 ± 0.038 | 0.73 ± 0.036 | 0.72 ± 0.051 | 0.68 ± 0.031 | 0.9002 | 0.62 ± 0.035 | 0.66 ± 0.035 | 0.62 ± 0.032 | 0.62 ± 0.026 | 1.0 |
| Bv (mm^2^) | 0.0031 ± 0.00081 | 0.00054 ± 0.00076 | 0.0 ± 0.0011 | 0.0 ± 0.00065 | 1.0 | 0.0 ± 0.0 | 0.0 ± 0.0 | 0.0 ± 0.0 | 0.0 ± 0.0 | N.A. |
| Mv (mm^2^) | 0.96 ± 0.026 | 0.91 ± 0.024 | 0.71 ± 0.034 | 0.74 ± 0.021 | 0.7943 | 0.76 ± 0.028 | 0.8 ± 0.028 | 0.71 ± 0.025 | 0.68 ± 0.021 | 0.8302 |
| Av (mm^2^) | 1.8 ± 0.057 | 1.6 ± 0.054 | 1.4 ± 0.076 | 1.4 ± 0.046 | 1.0 | 1.4 ± 0.054 | 1.5 ± 0.054 | 1.3 ± 0.049 | 1.3 ± 0.040 | 0.977 |
| Mv/Av (%) | 54 ± 1.0 | 55 ± 0.98 | 50 ± 1.4 | 52 ± 0.84 | 0.4499 | 55 ± 1.4 | 55 ± 1.4 | 54 ± 1.2 | 52 ± 1.0 | 0.9149 |
| Cv/Av (%) | 46 ± 1.0 | 45 ± 0.98 | 50 ± 1.4 | 48 ± 0.83 | 0.4342 | 45 ± 1.4 | 45 ± 1.4 | 46 ± 1.3 | 47 ± 1.0 | 0.9215 |
| Ct (um) | 190 ± 7.3 | 180 ± 6.8 | 190 ± 9.7 | 180 ± 5.8 | 0.7205 | 170 ± 7.6 | 170 ± 7.6 | 170 ± 6.9 | 170 ± 5.6 | 0.9954 |
| CSa (mm^2^) | 0.82 ± 0.038 | 0.73 ± 0.036 | 0.72 ± 0.051 | 0.68 ± 0.031 | 0.9002 | 0.62 ± 0.035 | 0.66 ± 0.035 | 0.62 ± 0.032 | 0.62 ± 0.026 | 1.0 |
| Vv (mm^2^) | 0.00012 ± 0.00045 | 0.00017 ± 0.00042 | 0.00069 ± 0.00059 | 0.0010 ± 0.00036 | 0.9624 | 0.000024 ± 0.00016 | 0.000048 ± 0.00016 | 0.00004 ± 0.00014 | 0.00044 ± 0.00012 | 0.1626 |
| CortBoneDensity (%) | 100 ± 0.062 | 100 ± 0.058 | 100 ± 0.082 | 100 ± 0.049 | 0.8842 | 100 ± 0.024 | 100 ± 0.024 | 100 ± 0.022 | 100 ± 0.018 | 0.1247 |
| TotalBoneDensity (%) | 46 ± 1.0 | 45 ± 0.97 | 50 ± 1.4 | 48 ± 0.83 | 0.4304 | 45 ± 1.4 | 45 ± 1.4 | 46 ± 1.3 | 47 ± 1.0 | 0.9215 |

Data presented as mean ± SD. Ct. V: cortical volume, Mv: medullary volume, Tt. Ar: total cross-section area inside the periosteal envelope, Ct. Ar: cortical bone area, Ct. Ar/Tt. Ar: cortical area fraction, Ct. Th: average cortical thickness, Po. V: total pore volume, Ct. Po: cortical porosity, p-value present the data between *Col1a1*^G643S/+^ placebo and 4PBA treatment analyzed by ANOVA followed by Tukey-Kramer post hoc test. N.A.: not applicable
