# Supplementary material for: Characterization of a Novel Col1a1G643S/+ Osteogenesis Imperfecta Mouse Model with Insights into Skeletal Phenotype, Fragility, and Therapeutic Evaluations
Source: Calcif Tissue Int. 2025 Jan 3;116(1):13. doi: 10.1007/s00223-024-01320-2 (PMC11698804; doi:10.1007/s00223-024-01320-2)
Supplement: Supplementary file 9 — Supplementary file9 (DOCX 17 KB) [file 223_2024_1320_MOESM9_ESM.docx]

Supplemental Table 8 Effect of the 4PBA treatment for three-point bending mechanical test of femur at 12 weeks

|  | Male | | | |  | Female | | | |  |
| --- | --- | --- | --- | --- | --- | --- | --- | --- | --- | --- |
|  | Wild type | | *Col1a1*^G643S/+^ | |  | Wild type | | *Col1a1*^G643S/+^ | |  |
|  | placebo  (n = 7) | 4PBA  (n = 8) | placebo  (n = 4) | 4PBA  (n = 11) | p value | placebo  (n = 5) | 4PBA  (n = 5) | placebo  (n = 6) | 4PBA  (n = 9) | p value |
| Maximum Load (N) | 16 ± 1.1 | 14 ± 1.0 | 14 ± 1.5 | 12 ± 0.89 | 0.6617 | 12 ± 1.2 | 13 ± 1.2 | 13 ± 1.1 | 12 ± 0.89 | 0.8029 |
| Maximum Displacement (mm) | 0.78 ± 0.12 | 0.87 ± 0.11 | 0.40 ± 0.16 | 0.66 ± 0.097 | 0.4908 | 0.92 ± 0.11 | 0.96 ± 0.11 | 0.44 ± 0.096 | 0.48 ± 0.079 | 0.9867 |
| Stiffness (N/mm) | 93 ± 6.7 | 89 ± 6.3 | 83 ± 8.9 | 74 ± 5.3 | 0.7807 | 73 ± 7.2 | 77 ± 7.2 | 76 ± 6.6 | 65 ± 5.4 | 0.5792 |
| Fracture energy (Nmm) | 9.4 ± 1.7 | 9.5 ± 1.6 | 3.9 ± 2.3 | 6.7 ± 1.4 | 0.7448 | 8.3 ± 1.1 | 10 ± 1.1 | 4.1 ± 1.0 | 3.9 ± 0.82 | 0.9993 |

Data presented as mean ± SD. p-value present the data between *Col1a1*^G643S/+^ placebo and 4PBA treatment analyzed by ANOVA followed by Tukey-Kramer post hoc test.
